# First Report of* Clostridium lavalense* Isolated in Human Blood Cultures

**DOI:** 10.1155/2016/7231805

**Published:** 2016-07-10

**Authors:** Richard Garceau, Christine Bourque, Louise Thibault, Jean-Charles Côté, Jean Longtin, Marc-Christian Domingo

**Affiliations:** ^1^Service de Microbiologie Médicale et Maladies Infectieuses, Centre Hospitalier Universitaire Dr. Georges-L.-Dumont, Moncton, NB, Canada E1C 2Z3; ^2^Laboratoire de Santé Publique du Québec, Sainte-Anne-de-Bellevue, QC, Canada H9X 3R5; ^3^Centre de Recherche en Infectiologie de l'Université Laval, Ville de Québec, QC, Canada G1V 4G2

## Abstract

An 88-year-old man was admitted to the hospital with worsening malaise, fever, and weakness. Anaerobic blood culture bottles revealed the presence of an anaerobic, Gram-positive sporulated bacillus. Empirical antibiotherapy with intravenous piperacillin-tazobactam was initiated. The patient defervesced after four days and was switched to oral amoxicillin on his 6th day of antibiotic therapy and later discharged from the hospital. Four months later, he had recovered. The bacterium was initially identified as* Clostridium butyricum* using anaerobic manual identification panel. 16S rRNA gene sequence and phylogenetic analysis showed the bacterium to be* Clostridium lavalense*, a recently described species with no previously published case of isolation in human diagnostic samples so far. This is the first report of* Clostridium lavalense* isolation from human blood cultures. Further studies are needed in order to elucidate the role of* Clostridium lavalense* in human disease and its virulence factors.

## 1. Introduction

*Clostridium lavalense* (Phylum Firmicutes, Order Clostridiales) is a recently described bacterial species [1]. It is Gram-positive, spore-forming, anaerobic, and rod-shaped and was originally isolated from human faecal specimens. Although the extent of its habitat is unknown, it is believed to be restricted to the mammalian gastrointestinal tract [1]. Its pathogenicity in humans or in other animals had never been reported.

Herein, we present the first reported isolation of* Clostridium lavalense* in human blood cultures.

## 2. Case Presentation

An 88-year-old man was admitted to the hospital with worsening malaise, fever, and weakness. The patient had a prior medical history of bilateral hip prosthesis, high blood pressure, and hypercholesterolemia. In the months preceding his admission, he had developed a right-sided hip pain. On the day of admission, his family had found him lying on the floor, too weak to walk. His blood pressure was 94/22 mmHg, heart rate 64 beats/min, and temperature 38.9°C. The examination of his head, neck, heart, lungs, and abdomen did not reveal anything abnormal. He had no wound nor sore. The mobilization of his right hip showed a limited range of motion without pain.

Laboratory analysis revealed leukocytosis with a white blood cell count of 15.3 × 10^9^/L (normal: 4 × 10^9^/L to 11 × 10^9^/L), a normal hemoglobin level of 135 g/L (normal: 132 g/L to 170 g/L), and platelet count of 155 × 10^9^/L (normal: 130 × 10^9^/L to 400 × 10^9^/L). The creatinine was at 151 *μ*mol/L (normal: 58 *μ*mol/L to 110 *μ*mol/L), aspartate aminotransferases (AST) were at 63 U/L (normal: 17 U/L to 59 U/L), creatine phosphokinase (CPK) was at 669 U/L (normal: 55 U/L to 170 U/L), C-reactive protein (CRP) was at 230 mg/L (normal <10 mg/L), and procalcitonin was at 1.1 ng/mL (normal <0.1 ng/mL). Four sets of aerobic and anaerobic blood cultures were collected on two consecutive days.

A preliminary diagnostic of septic arthritis of the patient's right hip was suspected and a computed tomography (CT) scan of the pelvis was ordered. It showed no asymmetry or fluid collection around the hip prosthesis. The chest X-ray was read as normal. The patient was kept under observation, without antibiotic therapy.

The four anaerobic blood culture bottles became positive after 29 hours of incubation. Their Gram stain revealed the presence of Gram-negative rods. The culture showed the bacillus to be anaerobic, Gram-positive, and sporulated, typical of a* Clostridium* species. No other organism was isolated. A commercial anaerobic manual identification panel (RapID ANA II System, Remel, Lenexa, KS, USA) identified the bacillus as* Clostridium butyricum*, with >99.9% of probability. However, the isolate could not be identified by matrix-assisted laser desorption ionization-time-of-flight mass spectrometry (MALDI-TOF MS) (Bruker, Madison, WI, USA), after a complete extraction. Both empirical antibiotherapy with intravenous piperacillin-tazobactam and the search for the source of infection were initiated. A CT scan of the abdomen and pelvis showed multiple stones in the gallbladder without cholecystitis and no ascites nor colonic masses. A transthoracic echocardiogram revealed an aortic valve sclerosis without vegetation. A colonoscopy revealed three benign polyps without any sign of colitis or cancer. The patient defervesced after four days of intravenous piperacillin-tazobactam. On the 6th day of antibiotherapy, he was switched to oral amoxicillin for eleven more days. He was discharged from the hospital still weak, using a walker. Four months later, he had recovered his strength and was able to walk with a cane. He had no relapse of fever nor worsening of his right hip pain.

### 2.1. Molecular Analysis and MICs of Selected Antibiotics

Due to the discordance in identification between the RapID ANA II System and MALDI-TOF MS, the bacterium was sent to the Laboratoire de Santé Publique du Québec (LSPQ) for further characterization. It was designated LSPQ-04253 and was identified by 16S rRNA sequencing using the BigDye Terminator v3.1 Cycle Sequencing Kit on an ABI 3130xl Genetic Analyzer (Applied Biosystems, Foster City, CA, USA) [2]. Its nucleotide sequence was determined and showed 99.2% identities with its orthologous sequence in* Clostridium lavalense* type strain CCRI-9842^T^. To identify the taxonomic neighbors of the clinical isolate, the 16S rRNA gene sequences were used for an initial BLAST search (http://www.ncbi.nlm.nih.gov/BLAST) against GenBank. Phylogenetic and molecular evolutionary analyses with closely related* Clostridium* species were performed on 1,344 nucleotides with MegAlign® version 10 (DNASTAR, Madison, WI).

**Figure 1:**
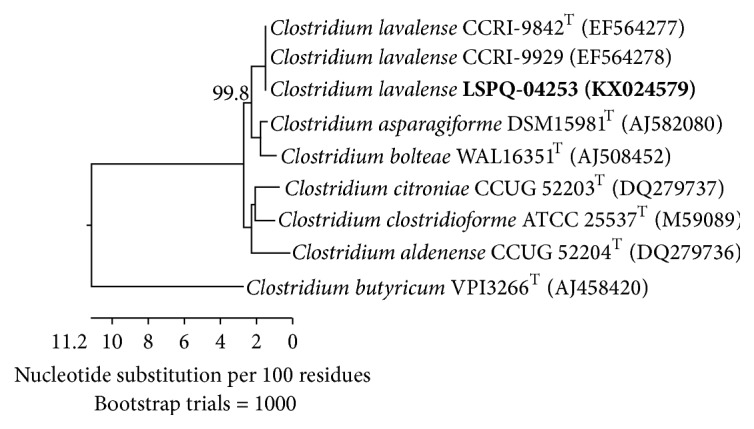
Neighbor-joining phylogenetic tree based on comparison of 16S rRNA gene sequences. Bootstrap percentage is shown at the node. The bar gives the number of nucleotide substitutions per 100 residues.

Multiple sequence alignment and phylogenetic analysis using 16S rRNA gene sequences of LSPQ-04253 (GenBank accession number KX024579) and those of* C. lavalense* CCRI-9842^T^ and CCRI-9929,* C. asparagiforme *DSM15981^T^,* C. bolteae *WAL16351^T^,* C. citroniae *CCUG52203^T^,* C. clostridioforme *ATC2553^T^,* C*.* aldenense *CCUG52204^T^, and* C. butyricum *VPI3266^T^ revealed that LSPQ-04253 clustered with the two* C. lavalense* strains and was distant from* C. butyricum *VPI3266^T^ (Figure 1). The antibiotic minimal inhibitory concentrations were determined using the agar dilution method [3] and E-test only for vancomycin and teicoplanin. The bacterium was susceptible to clindamycin, cefoxitin, meropenem, metronidazole, piperacillin-tazobactam, vancomycin, and teicoplanin and intermediate to penicillin (Table 1).

No* vanB* gene was found in isolate LSPQ-04253 as described in the type strain CCRI-9842^T^. The* vanB* gene is carried by a mobile genetic element (transposon Tn*5382*) in the type strain CCRI-9842^T^ that could be missing in isolate LSPQ-04253.

## 3. Discussion

Prior to the present case report, three* C. lavalense* strains had been reported worldwide, CCRI-9842^T^ and CCRI-9929, both from human faeces [1], and strain VE202-15 (GenBank accession number NZ_BAIC02000201.1). The case reported here presents the first isolation of* C. lavalense* in human blood cultures. This indicates that* C. lavalense* is a potential human pathogen.

Clostridia are present in the microbiota of the intestinal tract, the female genital tract, and the oral mucosa of humans [4]. A recent study suggested reduced abundance of* C. lavalense* in the faecal microbiota of patients with Crohn's disease [5].

Anaerobic bacteremia is uncommon, accounting for 5% of all positive blood cultures [6].* Clostridium* spp. account for only 10% of such anaerobic bacteremia [6]. Risk factors for* Clostridium* bacteremia include hemodialysis, intestinal malignancy, and inflammatory bowel disease [7]. Our patient had none of these predisposing factors. The point of entry of* C. lavalense* LSPQ-04253 into the patient's bloodstream remains unknown. The patient responded well to an empiric antibiotherapy with piperacillin-tazobactam, followed by amoxicillin. Susceptibility testing later confirmed the bacterium susceptibility to piperacillin-tazobactam. This finding is in agreement with a recent Canadian study on antimicrobial susceptibility of anaerobic bacteria in Ontario between 2010 and 2011. It showed that all non-*perfringens Clostridium* isolates were susceptible to piperacillin-tazobactam [8].

The bacterium was initially identified as* C. butyricum* by RapID ANA II but could not be identified using MALDI-TOF MS. It was clearly identified as* C. lavalense* by 16S rRNA gene sequencing and phylogenetic studies. This suggests that the bacteria involved in other cases of isolation of* C. butyricum* may have been misidentified and were in fact* C. lavalense*. A study on the epidemiology of* Clostridium* species bacteremia in Calgary between 2000 and 2006 showed that* C*.* butyricum* was involved in 4 of 138* Clostridium* bacteremia cases [9]. The* C*.* lavalense* 16S rRNA gene sequence belongs to the* Clostridium coccoides* rRNA group, the rRNA cluster XIVa of the genus* Clostridium* [1]. This cluster contains at least four very closely related and clinically relevant species:* C. bolteae*,* C. citroniae*,* C. clostridioforme*, and* C. aldenense*. Whether clinical cases which reported these four bacteria all identified the correct* Clostridium* species or failed to identify* C. lavalense* is unknown. Our work emphasizes the role of 16S rRNA gene sequencing and phylogenetic analysis in the proper identification of unusual pathogens and the importance of updating databases of conventional and new identification methods such as the RapID ANA II System and MALDI-TOF MS, respectively. The addition of* C*.* lavalense* in these databases will help in the proper identification of this bacterium in clinical cases. Further studies will be needed to define the role of* C. lavalense* in human disease and the presence of potential virulence factors.

## Figures and Tables

**Table 1:** MICs of selected antibiotics for *C. lavalense* LSPQ-04253.

MIC (*μ*g/mL)		
Antibiotic	*C*. *lavalense*	Interpretation
Clindamycin	2	S
Penicillin	1	I
Cefoxitin	4	S
Meropenem	0.5	S
Metronidazole	0.125	S
Piperacillin-tazobactam	1/4	S
Vancomycin	0.5	S
Teicoplanin	1	S

S: susceptible; I: intermediate.
